# Robust mean shift filter for mixed Gaussian and impulsive noise reduction in color digital images

**DOI:** 10.1038/s41598-022-19161-0

**Published:** 2022-09-02

**Authors:** Damian Kusnik, Bogdan Smolka

**Affiliations:** Faculty of Automatics, Electronics and Computer Science, Silesian University of Technology, Akademicka 16, 44-100 Gliwice, Poland

**Keywords:** Mathematics and computing, Computer science

## Abstract

Noise reduction is one of the most important topics of digital image processing and despite the fact that it has been studied for a long time it remains the subject of active research. In the following work, we present an extension of the Mean Shift technique, which is efficiently reducing the Gaussian noise, so that it is able to cope with the impulsive disturbances. Furthermore, the elaborated technique can be applied to enhance the images corrupted by a mixture of strong Gaussian and impulsive noise, severely decreasing the quality of color digital images. By means of our approach, which is based on a novel similarity measure between a pixel and a patch located in the center of the processing block, even heavily disturbed images can be effectively restored, which enables the success of further stages of the image processing pipeline. We evaluate the efficiency of the proposed method using a publicly available database of test color images and compare the restored images applying a set of standard quality metrics with the results delivered by state-of-the-art denoising methods. Additionally, we compare our method with the Medoid and Quick Shift techniques, accelerating the original Mean Shift algorithm, in terms of objective quality criteria and computational complexity. The results of the performed experiments indicate that the proposed technique is superior to the widely used denoising techniques and can be used as a robust extension of the Mean Shift procedure. In the paper, a particular emphasis is placed on the ability of the presented algorithm to preserve and enhance image edges. The performed experiments evaluated with the use of the Pratt’s index, quantitatively confirm the superiority of the proposed design over the Mean Shift and standard denoising methods. The preservation of edges and even their sharpening is a very important feature of our algorithm whereas the final goal is segmentation, playing a crucial role in various computer vision tasks. The proposed algorithm is intended for the mixed noise reduction in color images, but it can be also applied in multispectral imaging and clustering of multidimensional data. To enable the comparison of our method with the standard denoising techniques and to help applying it in other image processing fields, we made its code freely available.

## Introduction

Noise reduction has been one of the paramount topics in digital image processing for decades. Over recent years, mainly due to the ubiquity of multimedia devices and popular applications for capturing, uploading and sharing color images through social networks, research focused on improving the quality of digital images has rapidly gained in importance.

The resolution of smartphones, webcams, video recorders and other devices that are used for the acquisition of visual information has notably increased and despite the fact that less and less light falls on the photosensitive sensors, images are expected to be of an excellent quality. Therefore, the development of effective and fast algorithms to reduce noise, improve contrast, color saturation and increase tonal dynamics is gaining more and more significance^1–3^. The demand for new, efficient noise reduction algorithms is also influenced by novel multi-channel imaging techniques, particularly in medicine and biology, astronomy, earth observation and industrial applications. In all imaging modalities, noise suppression plays a crucial role as in general, improving the quality of images by reducing distortion levels, implies the success of further processing and analysis^4,5^.

In the undermentioned paper we focus on the Mean Shift (MS) technique, which is an effective method of reducing the impact of noise-induced distortions that can smooth out the noise while preserving image edges. Owing to the ability of MS to flatten the color images, it is also used as a pre-processing stage in image segmentation tasks. This method works successfully when enhancing images corrupted by Gaussian noise, but it severely fails when impulsive noise is also present, as the outlying pixels are being preserved. Hence, to exploit the capabilities of the MS method, very often a suitable impulsive noise removal technique has to be applied firstly and afterwards, the MS is performed to reduce the remaining Gaussian noise component.

To alleviate the problems caused by the impulsive noise corrupting the images, we propose a robust modification of the standard MS technique. Our method may work directly on the noisy color images distorted by mixed impulsive and Gaussian noise without the need to remove the impulses at first and then, reduce the remaining Gaussian noise component. In addition, it offers very satisfying denoising results. As the proposed Robust Mean Shift (ROMS) can be directly applied to restore images corrupted by even strong mixed Gaussian and impulsive noise, it can be used in various practical imaging tasks. The described technique has been validated on a database available for download at http://denoising.net/ and also accessible as electronic supplementary material^6^, containing a variety of color test images contaminated with different noise intensity levels, and the obtained results show that it significantly outperforms the classical MS algorithm and its modifications. It is worth stating at this point that the comparison of the proposed robustified MS with state-of-the-art techniques also confirms its satisfying denoising performance. Using the elaborated technique, noise is efficiently suppressed even in heavily corrupted images, edges are substantially sharpened and details are retained.

In this work we also prove that the application of the pixel to patch similarity concept introduced in^7,8^ allows to successfully restore color images corrupted by mixed impulsive and Gaussian noise using the MS based procedure without the need of any preprocessing. We provide the results of simulations which can guide the users while setting the parameters of the new filter. We also show that the proposed denoising framework is able to enhance image edges that can be crucial in many practical applications.

The rest of the paper is structured as follows. First, in “Related work” we briefly describe previous techniques focused on the reduction of the mixture of Gaussian and impulsive noise. Afterwards, in “Mean shift and its fast modifications” the Mean Shift technique is presented and we provide a description of its modifications, which main aim is to decrease its computational complexity. In “Robust mean shift ” we put forward the structure of the proposed Robust Mean Shift (ROMS) algorithm and in “Efficiency evaluation and comparison with existing filtering designs” we evaluate the impact of the proposed filter parameters on its efficiency when restoring color images affected by mixed impulsive and Gaussian noise of varying intensity. We also compare the efficiency of the ROMS filter with various standard filtering methods using image quality measures as well as the Pratt’s Figure of Merit. Finally, we draw some summarizing conclusions and discuss future work directions.

## Related work

Various kinds of noise degrade the quality of digital images, introducing distortions of its original content. Common noise sources include malfunctioning pixel elements in the camera sensors, thermal noise of the photosensitive matrix, shot noise caused by fluctuations in the photon flux, faulty memory locations or bit errors in hardware, timing errors in analog-to-digital converters, noise generated by electromagnetic interference and atmospheric turbulence as well as errors caused by imperfect optics and transmission. The resulting image degradations are frequently modelled as a mixture of additive white Gaussian noise, mainly responsible for dark and shot currents, and impulsive noise generating pixels with random channel intensity values^3,9–11^.

Standard Gaussian noise filtering schemes estimate the intensity of the processed pixel or its color channel values, considering its similarity to surrounding pixels belonging to a local processing block. The popular Bilateral Filter (BF)^12,13^ takes into account the radiometric and topographic closeness between pixels and can effectively suppress image distortions while preserving edges. Nonetheless, it fails in the presence of impulsive disturbances, as the corrupted pixels are being preserved. The ability of the bilateral filter to enhance edges was extended by incorporating a guidance image, which can be the processed image itself or an output of a robust filter capable of removing impulsive noise^14,15^, so that the mixed noise can be better suppressed.

The efficiency of BF was considerably increased by the use of additional information about the structure of a pixel neighborhood. The Non-Local Means filter (NLM)^16^ compares pixel patches instead of single pixels and by using a local measure of similarity between the patches, image details and edges are better preserved. Though, impulsive noise severely distorts the distance between the patches and, since as in the BF, pixels are also compared to themselves, it results in the inability of NLM to eliminate outlying pixels.

The impulsive noise is also affecting the efficiency of the Block-Matching and 3D Filtering, (BM3D)^17,18^ which is exploiting the image local sparse representation in the transform domain^19–23^ and dampen the Gaussian noise operating on a 3D stack of the local patches from the sliding filtering block, applying a collaborative filtering-based shrinkage strategy. The Anisotropic Diffusion filter^24^ also cannot handle the impulses, as the local gradients between an impulsive pixel and its neighbors are high, which remarkably slows down the diffusion process and preserves the outlying pixels^25^.

To alleviate the above-mentioned problems, numerous image restoration approaches were designed to first remove impulsive noise and then, to apply an efficient filter intended for the reduction of the Gaussian noise component^26–30^. The rationale behind such an approach is that the nature of the two noise degradation types is distinct and they are easier to manage when considered separately. Nevertheless, the Gaussian noise, through its masking effect, diminishes the accuracy of impulse detection methods, and in the following processing stage the remaining noise, which characteristics is generally far from the assumed Gaussian distribution, is not satisfactorily attenuated, with visible artifacts and some amount of impulses still unfiltered.

The Mean Shift technique, which extension is the subject of the following paper, can be used to detect impulsive noise. As the MS preserves the outlying pixels, they can be removed in the successive filtering step and the remaining image disturbances can be reduced with a suitable standard technique designed to cope with Gaussian noise^31–33^. In^34^ the authors proposed firstly detecting the impulses using the Adaptive Center-Weighted Median Filter (ACWMF) and replacing them with Adaptive Median Filter (AMF). Subsequently, the BM3D filter is applied to smooth out the Gaussian noise component and finally, the previously detected outliers are corrected using an inpainting method based on the median filter. The ACWMF and AMF impulsive noise filters were also used to detect the outliers when denoising the image using blind inpainting methods^35–39^ and were applied in^35^ to ensure the robustness of a method based on sparse image representation model.

Another technique^40^ combines the direction weighted median filter aimed at eliminating impulsive noise with the BM3D. The filtering result is further refined, detecting the remaining noise, which was not removed in the second processing stage. In^41,42^ the impulsive noise was removed with an adaptive averaging filter and the remaining distortions were suppressed with BM3D. In^43^ the Robust Outlyingness Ratio, which is a local statistic capable of efficiently detecting outliers, was used to remove impulsive noise disturbances and then the NLM filter was applied, adapting its parameters to the remaining noise characteristic. In^44^ the impulses were first detected by sorted quadrant median vector^28^ and the unfiltered noise was smoothed out by BF, which parameters were tuned on the basis of an estimation of the mixture noise composition and its intensity. The method of mixed noise reduction described in^45^ firstly detects the impulses and the subsequent stages use filters utilizing the PCA technique.

The approach proposed in^30^ consists of a fuzzy impulsive noise removal filter followed by additive noise reduction and a final postprocessing step. Once the impulsive noise is suppressed, a method based on sparse representation and 3D-processing performed with the use of DCT is applied. In the end, BF and an edge restoration technique is employed. An approach based on fuzzy logic was elaborated to cope with the problem of impulsive noise detection in^46^. The outlier detection scheme, which compares the central pixel with its neighbors, is designed to prevent the filtering of noise-free image pixels. Then, a weighted averaging scheme incorporating the measure of pixel impulsiveness is used to suppress the noisy pixel. A fuzzy-based switching technique for impulse detection was also devised in^47^. The proposed scheme builds fuzzy membership functions based on the local pixel similarity to its neighborhood and the results of median processing.

The efficiency of the two-stage approaches are generally not satisfactory as the methods require setting proper values of parameters dependent on the mixed noise characteristics and intensity. They are also computationally more demanding and are prone to produce visible artifacts. Therefore, many robust methods capable of suppressing in one step, both impulsive and Gaussian noise, have been developed. Primarily, these methods combine techniques of outlier detection with existing filtering designs so that the undesired influence of impulses can be eliminated or at least diminished.

A reliable statistic used to estimate pixel impulsiveness is the Rank Ordered Absolute Difference, (ROAD) which calculates the sum of distances between a pixel and its most similar neighbors from a filtering window^48,49^. The ROAD statistic was applied in the construction of the Trilateral Filter, which incorporates it into the BF framework^50–53^. The extension of the ROAD statistics was also applied for impulse detection in the method proposed in^54^, which restores the corrupted pixels with an interpolation scheme based on the radial basis function.

A robust modification of the NLM filter was proposed in^55^. As the patches are contaminated by impulsive noise, the noisy pixels are assigned a weight using the ROAD measure^56,57^. The weights are utilized when calculating the similarities between the patches, which are used to build the weighted average of pixels from a processing block. The proposed approach proved to be more effective when the spatial difference is considered, like in the BF^58^. The authors of^59^ propose applying in the first stage the traditional NLM technique and then, removing the remaining distortions again with NLM, which coefficients depend on the difference between the pre-denoised result and the noisy image, and also take into account the result of the first denoising step.

The patch-based method was also applied in^60^. First, the intensity of the mixed noise is estimated using the ROAD measure, then the central patch of the processing block is compared with all other patches and the most similar are chosen and finally, a given pixel is denoised by applying a maximum likelihood estimator. The extension of the ROAD statistics to color image processing was also developed in^61,62^. The ROAD statistics, obtained using various kinds of Minkowski distance in the RGB color space, are used both, as a measure of noise distortion and also, as its similarity to neighboring pixels and are exploited in the construction of a fuzzy filter which parameters are tuned to the noise characteristic. Another simple fuzzy filtering approach is based on the weighted vector median filter and the similarity between pixels expressed by ROAD^63^. The authors of^64^ proposed a fuzzy filter which can remove mixed noise in color images. The elaborated method relies on fuzzy rules based on the aggregated distances between the processed pixel and its neighbors.

A wavelet multi-scale analysis, combined with the local averaging filter was applied in^65^ and in^66^ an edge-preserving image denoising framework based on wavelet transform was also proposed. To successfully suppress noise distortions, a locally adaptive patch-based thresholding scheme was applied. The discrete wavelet transform coupled with the double window median filter was also used in^67^. The method utilizes row and column windows to perform median filtering and the wavelet coefficients are thresholded to suppress the noise-induced disturbances. Another technique developed in^68^ first smooths the image with a Gaussian or bilateral filter and then, examines the difference between the noisy and filtered images. To suppress the noise and retain the image details, the wavelet decomposition and thresholding were performed. The final restoration is achieved by using wavelet reconstruction, which efficiently estimates the image noise component.

An algorithm using clustering-based sparse representation exploiting both sparsity and non-local self-similarity was designed in^69^. The performed experiments confirm the competitive performance of the new technique which can be applied to video denoising. In^70^ the image denoising is formulated as an optimization problem that is solved iteratively by a weighted basis pursuit in the closed affine subspace. The reconstruction of the extracted noisy patches is performed by sparse representation using two dictionaries built with the DCT. The weighted encoding with the sparse non-local regularization technique (WESNR) was also applied in^71^ to cope with mixed noise. The noise-corrupted image patches were encoded over a set of pre-learned local PCA dictionaries and coding residuals were weighted adaptively to evaluate the pixel corruption measure. Additionally, both image sparsity and non-local self-similarity priors were combined into a single sparse regularization term. Another method depicted in^72^ first performs the initial denoising with the filter described in^55^. Then, the image pixels are classified as corrupted by impulsive or Gaussian noise and the final output is obtained using a variational approach. A method based on the total variation^73–75^ with $\ell _0$-norm fidelity was described in^76^. Although designed for impulsive noise, it can efficiently reduce various mixtures of noise models, too.

Impulsive noise can be also detected using the methods developed within the framework of mathematical morphology^77–80^ which enables the construction of two-stage filtering designs. In^81^ various impulsive noise detectors based on morphological operators were evaluated and the remaining noise was suppressed using morphological smoothers. A combination of the fuzzy approach to noise reduction with morphological operations was presented in^82^. The experiments revealed that the elaborated approach yields very promising results for highly contaminated images.

This brief review of the literature shows that mixed noise reduction methods can be divided into two classes. The algorithms belonging to the first class detect the impulses first and then reduce the remaining Gaussian noise. The second class of algorithms allows for simultaneous removal of both types of disturbances by introducing robust mechanisms of the suppression of outliers introduced by impulsive noise.

The analysis of the denoising algorithms shows that modifications of methods which are intended to reduce Gaussian noise, generally achieve good efficiency. Therefore, in this work we present a modified Mean Shift algorithm based on the robust pixel to patch similarity measure, which can be used to extend other Gaussian noise reduction methods such as BF, NLM or BM3D to diminish their susceptibility to impulsive disturbances.

## Mean shift and its fast modifications

The Mean Shift is a powerful nonparametric iterative technique which is used for finding the local modes of a given density function. It was introduced in^83^ and has been further investigated by the authors of^84^, however its immense popularity in image processing is due to the seminal work presented in^85^. This mode-seeking algorithm is based on the widely used kernel smoothing technique^86^ and features some similarity with the popular k-means clustering algorithm and the image smoothing approaches based on bilateral filtering and nonlinear diffusion^25,87,88^. Therefore, it is frequently applied for edge-preserving noise reduction^89^, object tracking and color image, and video segmentation^90–92^.

The MS technique is fairly effective in enhancing color images disturbed by the low-intensity Gaussian noise. It may smooth out the undesired noise component while minimizing the loss of sharpness of the image edges. Alas, this technique fails completely when the image is also corrupted by heavy-tailed noise, as the introduced impulses are treated as local modes and can be removed only when depleting the image contrast, which leads to its unacceptable blurry appearance. The inability of MS to remove impulses can be used for their detection, which enables their further treatment by a selected interpolation method^32,93,94^. To circumvent the effect of outlying pixels preservation, the impulses can be first removed using a method adapted to the impulsive noise intensity and the remaining pixels are later restored using the MS iterative scheme^95^.

Let us recall the structure of the MS technique, assuming a sample $\varvec{X}=\{\varvec{x}_1, \ldots , \varvec{x}_n\}$ which consists of *n* multivariate observations $\varvec{x}_i$, ($i=1,\ldots , n$) in the *l*-dimensional space $\mathbb {R}^l$. The multivariate density estimator $f(\varvec{x})$ calculated at observation $\varvec{x}$ is defined using a kernel $\Phi (\varvec{x})$ which is a bounded and symmetric function defined as^85,96^

1 $$\begin{aligned} f(\varvec{x})= \frac{1}{n \sigma ^l} \sum _{i=1}^{n} \Phi \left( \frac{ \varvec{x} -\varvec{x}_i}{\sigma } \right) , \end{aligned}$$

where $\sigma$ is the bandwidth (smoothing parameter).

In practice, the radially symmetric kernel defined as $\Phi (\varvec{x})=c_{\phi ,l}\cdot \phi (\Vert \varvec{x} \Vert ^2)$ is used and we obtain

2 $$\begin{aligned} f(\varvec{x})= \frac{c_{\phi ,l}}{n\sigma ^l} \sum _{i=1}^{n} \phi \left( \left\| \frac{ \varvec{x} -\varvec{x}_i}{\sigma } \right\| ^2 \right) , \end{aligned}$$

where $\Vert \cdot \Vert$ denotes the Euclidean norm, $\phi$ is a function called profile and $c_{\phi ,l}$ is the normalizing factor. The popular choice of the profile is $\phi (x)=\exp \left( -x/2 \right)$, which provides the Gaussian kernel $\Phi _{\text {G}}(\varvec{x})=(2\pi )^{-\frac {l}{2}} \exp \left( - \Vert \varvec{x} \Vert ^2/2 \right) .$

Introducing the function $\psi (x)=-\phi ^{'}(x)$ we can define a kernel $\Psi (x)=c_{\psi ,l}\, \psi \left( \Vert \varvec{x} \Vert ^2 \right)$, with $c_{\psi ,l}$ being a normalizing constant. The kernel $\Psi$ is called a shadow of $\Phi$ and thus the Epanechnikov profile is a shadow of a uniform (flat) profile $\psi _{\text {U}}(x)= 1$ if $\Vert x\Vert \le 1$ and 0 otherwise, while the Gaussian kernel and its shadow have the same form.

The gradient of the density estimator $f(\varvec{x})$ defined in (2) is thus given by

3 $$\begin{aligned} \begin{aligned} \nabla f(\varvec{x})= C \sum _{i=1}^{n} (\varvec{x}-\varvec{x}_i)\, \phi ^{'}\left( \frac{ \Vert \varvec{x} -\varvec{x}_i\Vert ^2}{\sigma ^2} \right) , \end{aligned} \end{aligned}$$

where $C=2c_{\phi ,l}/n\sigma ^{l+2}$ is a constant. As $\phi ^{'}(x)=-\psi (x)$ we obtain

4 $$\begin{aligned} \begin{aligned} \nabla f(\varvec{x})= C \sum _{i=1}^{n} (\varvec{x}_i-\varvec{x})\, \psi \left( \frac{ \Vert \varvec{x} -\varvec{x}_i\Vert ^2}{\sigma ^2} \right) = C \left[ \sum _{i=1}^{n} \varvec{x}_i \psi \left( \frac{ \Vert \varvec{x} -\varvec{x}_i\Vert ^2}{\sigma ^2}\right) - \varvec{x} \left\{ \sum _{i=1}^{n} \psi \left( \frac{ \Vert \varvec{x} -\varvec{x}_i\Vert ^2}{\sigma ^2}\right) \right\} \right] , \end{aligned} \end{aligned}$$

which can be rewritten as

5 $$\begin{aligned} \begin{aligned} \nabla f(\varvec{x})= C \left\{ \sum _{i=1}^{n} \psi \left( \frac{ \Vert \varvec{x} -\varvec{x}_i\Vert ^2}{\sigma ^2}\right) \right\} \left\{ \frac{\sum _{i=1}^{n} \varvec{x}_i\, \psi \left( \frac{ \Vert \varvec{x} -\varvec{x}_i\Vert ^2}{\sigma ^2}\right) }{\sum _{i=1}^{n} \psi \left( \frac{ \Vert \varvec{x} -\varvec{x}_i\Vert ^2}{\sigma ^2}\right) } -\varvec{x} \right\} . \end{aligned} \end{aligned}$$

The first term of (5) is proportional to the density estimation calculated using the kernel $\Psi$ and the second one is called *Mean Shift* denoted as $\varvec{m}(\varvec{x})$

6 $$\begin{aligned} \varvec{m}(\varvec{x})= \frac{\sum _{i=1}^{n} \varvec{x}_i\, \psi \left( \Vert \varvec{x} - \varvec{x}_i\Vert ^2/\sigma ^2 \right) }{\sum _{i=1}^{n} \psi \left( \Vert \varvec{x} -\varvec{x}_i\Vert ^2/\sigma ^2\right) } -\varvec{x} =\varvec{y}-\varvec{x}, \end{aligned}$$

where $\varvec{y}$ is a weighted mean of $\{\varvec{x}_i\}$ and the weights are determined by the appropriate values of the $\psi$ function. The mean shift vector $\varvec{m}(\varvec{x})$, which is the difference between the normalized weighted average obtained using the kernel $\psi$ and the vector $\varvec{x}$, is oriented in the direction of the gradient of the density function, or in other words, points towards its maximum increase.

The mean shift procedure works in an iterative way. The observation $\varvec{x}$ is being shifted to $\varvec{y}$ as shown in (6) and thereby, we can formulate the iterative mode finding scheme as^85,97^

7 $$\begin{aligned} \varvec{x}^{(t+1)}= \frac{\sum _{i=1}^{n} \varvec{x}_i\, \psi \left( \Vert \varvec{x}^{(t)} -\varvec{x}_i\Vert ^2/\sigma ^2 \right) }{\sum _{i=1}^{n} \psi \left( \Vert \varvec{x}^{(t)} -\varvec{x}_i\Vert ^2/\sigma ^2 \right) }, \end{aligned}$$

where $t\ge 1$ denotes the iteration number and $\varvec{x}^{(1)}$ is the starting point. This iterative technique converges to a local mode^85^ and the process is terminated when the magnitude of the mean shift vector satisfies $\Vert \varvec{m}(\varvec{x})\Vert <\epsilon$, where $\epsilon$ is a small scalar value.

The mean shift procedure defined by (7) can be formulated as locally weighted least squares estimator^98–101^

8 $$\begin{aligned} \varvec{x}^{(t+1)}=\mathop {arg\,min}\limits _{\varvec{x}} \sum _{i=1}^{n} \Vert \varvec{x}-\varvec{x}_i\Vert ^2 \psi \left( \frac{\Vert \varvec{x}^{(t)} -\varvec{x}_i \Vert ^2 }{\sigma ^2 }\right) . \end{aligned}$$

Calculating the gradient of the minimized expression and equating it to zero we obtain expression (7)^100^ and in the iteration $(t+1)$, a point $\varvec{x}=\varvec{x}^{(t+1)}$ is being found, which minimizes the cost function $\sum _{i=1}^{n} \Vert \varvec{x}-\varvec{x}_i\Vert ^2 \psi \left( \Vert \varvec{x}^{(t)} -\varvec{x}_i \Vert ^2 /{\sigma ^2 }\right)$. As can be readily noticed, this procedure is well known from the theory of M-estimators. The process of finding the successive points $\varvec{x}^{(t)}$ is time-consuming as many iterations can be needed to obtain the $\epsilon$-convergence and in each iteration the distances between $\varvec{x}^{(t)}$ and all points $\{\varvec{x}_i\}$ need to be computed.

To speed up the process of finding the local modes, the Medoid Shift algorithm, which is based on the iterative procedure formulated in (8), has been proposed^100,102^. This modification assumes that only the points that belong to the set $\varvec{X}$ can be taken as successive observations that minimize the cost function, and the trajectory is determined by

9 $$\begin{aligned} \begin{aligned} \varvec{x}^{(t+1)}= \mathop {arg\,min}\limits _{\varvec{x}^{(t)}\in \{\varvec{x}_1,\ldots ,\varvec{x}_n\}}\; \;\sum _{i=1}^{n} \Vert \varvec{x}^{(t)}-\varvec{x}_i\Vert ^2 \psi \left( \Vert \varvec{x}^{(t)} -\varvec{x}_i \Vert ^2 /{\sigma ^2 }\right) , \end{aligned} \end{aligned}$$

or by

10 $$\begin{aligned} \begin{aligned} \varvec{x}^{(t+1)}= \mathop {arg\,min}\limits _{\varvec{x}^{(t)}\in \{\varvec{x}_1,\ldots ,\varvec{x}_n \}} \;\sum _{i=1}^{n} d^2(\varvec{x}^{(t)},\varvec{x}_i) \cdot \psi \left( d^2(\varvec{x}^{(t)}, \varvec{x}_i)/\sigma ^2 \right) , \end{aligned} \end{aligned}$$

where $d^2(\varvec{x}^{(t)},\varvec{x}_i)$ is the squared dissimilarity measure between the corresponding vectors, equal to $\Vert \varvec{x}^{(t)}-\varvec{x}_i\Vert ^2$ when the Euclidean norm is used and $\varvec{x}^{(1)}$ is again the starting point.

As a result, the trajectory of $\varvec{x}^{(t)}$ obtained in successive iterations is constrained to pass only through the points that belong to the initial set $\varvec{X}$. Although much faster than the original MS technique, this algorithm is also time-consuming as all the distances from the current position $\varvec{x}^{(t)}$ to the points $\varvec{x}_1,\ldots ,\varvec{x}_n$ belonging to $\varvec{X}$ have to be calculated to determine the next iteration output $\varvec{x}^{(t+1)}$. Notwithstanding, the computation of the distances between the points belonging to $\varvec{X}$ has to be performed only once and when stored they can be reused in the iteration process described by (10).

Another approach aiming to speed up the iterative mode finding procedure was proposed in^99^. The authors put forward a method named Quick Shift (QS), which is defined by

11 $$\begin{aligned} \varvec{x}^{(t+1)}=\mathop {arg\,min}\limits _{\varvec{x}_i:\, f(\varvec{x_i})>f( \varvec{x}^{(t)} ) } \!\!\! d^2(\varvec{x}^{(t)},\varvec{x}_i), \end{aligned}$$

where $f(\varvec{x}_i)$ and $f(\varvec{x}^{(t)})$ are the values of the density function defined by (1) at $\varvec{x}_i$ and $\varvec{x}^{(t)}$ and $d^2$ is again a squared dissimilarity measure.

Using the Quick Shift, each initial point is moved to its nearest neighbor, for which the density function is increased and this process is repeated. As might be observed, if a global mode is present, then it is the final point of every trajectory that originates from the initial starting point $\varvec{x}^{(1)}$. Therefore, some constraints regarding the maximum value of dissimilarity between the successive points in a trajectory can be predefined, so that the paths created through the iterative scheme are terminated before the global mode is reached.

In regard to color images, the mean shift procedure operates in 5-dimensional space, ($l=5$). Two dimensions describe the pixel position, and another three are needed for the RGB color channels. The spatial and radiometric dimensions expressed by vectors $\varvec{\xi }$ and $\varvec{\eta }$ have to be treated in a different way, which is reflected in the definition of the radial kernel, which is composed of two parts

12 $$\begin{aligned} \Phi (\varvec{x})=\Phi _{\text {s}}(\varvec{\xi }/\sigma _{\text {s}}) \cdot \Phi _{\text {r}}(\varvec{\eta }/\sigma _{\text {r}}), \end{aligned}$$

where $\sigma _{\text {s}}$ and $\sigma _{\text {r}}$ are kernel bandwidths (smoothing parameters) corresponding to the spatial (s) and radiometric (r) components.

Due to the properties of natural images, the correlation between pixels decreases with their topological distance on the image domain. This is incorporated into the spatial kernel, which is mostly a Gaussian function or is simplified by a flat kernel. Usually, the mean shift operates using a square processing block $\mathcal {B}$ of size $(2r+1)\times (2r+1)$, where the radius *r* determines the ability of MS to determine the local modes, so that it can be viewed as a parameter influencing the extrema finding resolution. The processing block $\mathcal {B}$ contains $N=(2r+1)^2$ pixels which are indexed by $j=1, \ldots , N$.

The second parameter $\sigma _{\text {r}}$ influences the role of the pixel color components. If its value is small, only similar pixels are considered, whereas for large values, owing to oversmoothing, the local modes cannot be distinguished. The role of the size of the processing block is also clear in the case of the QS algorithm, for which the limited size of the processing block can prevent the digital paths to reach the global mode.

The MS algorithm iteratively calculates the new position $\varvec{\xi }_i^{(t+1)}$ of the center of a processing block $\mathcal {B}_i^{(t+1)}$ and its updated RGB values expressed by $\varvec{\eta }_i^{(t+1)}$. The index *i* determines the position of a central pixel of the processing block. The procedure starts from the pixel position $\varvec{\xi }_{i}^{(1)}$ at the center of the initial block $\mathcal {B}_i^{(1)}$ which consists of pixels indexed by $j=1,\ldots ,N$, where $N=(2r+1)^2$ is the number of pixels in the sliding block. The RGB components of the initial pixel at location $\varvec{\xi }_i^{(1)}$ are contained in the vector $\varvec{\eta }_i^{(1)}$ and the same procedure is applied to each image pixel $\varvec{x}_i$. Thus, the MS performs a series of iterations

13 $$\begin{aligned} \varvec{\xi }_i^{(t+1)}= \frac{\sum _{j=1}^N w_j \cdot \varvec{\xi }_j }{\sum _{j=1}^N \, w_j}, \quad \varvec{\eta }_i^{(t+1)}= \frac{ \sum _{j=1}^N w_j \cdot \varvec{\eta }_{j} }{\sum _{j=1}^N \, w_j } , \end{aligned}$$

where

14 $$\begin{aligned} w_j=\exp \left\{ - \frac{\Vert \varvec{\eta }_i^{(t)}-\varvec{\eta }_j\Vert ^2}{2\sigma _{\text {r}}^2}\right\} \cdot \exp \left\{ \frac{\Vert \varvec{\xi }_i^{(t)}-\varvec{\xi }_j\Vert ^2}{2 \sigma _{\text {s}}^2} \right\} , \end{aligned}$$

and $\sigma _{\text {r}}$, $\sigma _{\text {s}}$ are smoothing parameters and each block $\mathcal {B}_i^{(t)}$ contains pixels indexed from $j=1$ to *N*.

The algorithm stops in iteration $(t+1)$ when $\Vert \varvec{x}^{(t+1)} -\varvec{x}^{(t)} \Vert <\epsilon$, where $\epsilon$ is a predefined small value, (set in our experiment at $10^{-3}$). When this condition is satisfied, the local mode is reached and the magnitude of the mean shift vector is very close to 0. Then the final value of $\varvec{\eta }_i^{(t)}$ is assigned to the pixel at the initial position $\varvec{\xi }_i$. The structure of the standard MS algorithm is presented using a pseudocode in Alg. 1.
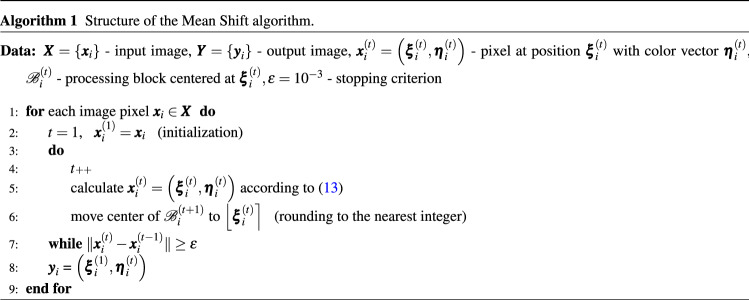


## Robust mean shift

The new approach to the problem of mixed Gaussian and impulsive noise suppression in color images is based on the previously discussed MS technique and the recently introduced Robust Local Similarity Filter (RLSF)^7,8^, which exhibits a very satisfying ability to reduce mixed Gaussian and impulsive noise in color images. The RLSF is based on the bilateral filter and a modified ROAD statistic.

In order to process an image pixel $\varvec{x}_i$ and calculate the filter output $\varvec{x}_i^{'}$, a weighted average of pixels from a block $\mathcal {B}_i$ centered at $\varvec{x}_i$ is computed. The weighting function utilizes the Robust Similarity Measure (RSM) defined as

15 $$\begin{aligned} R(\varvec{x}_j, \mathcal {W}_i)=\frac{1}{\alpha } \sum _{k=1}^{\alpha } d^2_{j(k)}, \end{aligned}$$

where $d_{j(k)}$, $k=1,\ldots ,9$, is the *k*-th smallest Euclidean distance between vector $\varvec{x}_j$ from the processing block $\mathcal {B}_i$ and the pixels from a window $\mathcal {W}_i$ of size $3 \times 3$ at the center of this block and $\alpha \in [1,9]$ is a parameter which denotes the number of closest neighbors taken for the average.

The RLSF filter output is defined as

16 $$\begin{aligned} \varvec{x}_i^{'}=(\lfloor \varvec{\xi }_i^{'}\rceil ,\varvec{\eta }_i^{'}),\quad \text {where} \quad \varvec{\xi }_i^{'}= \frac{ \sum _{j=1}^N \omega _j \cdot \varvec{\xi }_j }{ \sum _{j=1}^N \omega _j}, \quad \varvec{\eta }_i^{'}= \frac{ \sum _{j=1}^N \omega _j \cdot \varvec{\eta }_j }{ \sum _{j=1}^N \omega _j}, \end{aligned}$$

with

17 $$\begin{aligned} \begin{aligned} \omega _j= \exp \left( -\frac{R(\varvec{x}_j, \mathcal {W}_i)}{2\sigma ^2} \right) = \exp \left( - \frac{1}{2\alpha \sigma ^2} \sum _{k=1}^{\alpha } d^2_{j(k)} \right) , \end{aligned} \end{aligned}$$

where $\varvec{x}_j$, $j=1,\ldots , N$, are the pixels in the processing block $\mathcal {B}_i$, $d_{j(k)}$ is the Euclidean distance between $\varvec{x}_j$ and the central pixel $\varvec{x}_i$ of $\mathcal {B}_i$ and $\lfloor \cdot \rceil$ denotes rounding to the closest integer.

Thereby, only those pixels from the block $\mathcal {B}_i$, which are most similar to the small central window $\mathcal {W}_i$ are taken to the weight computation process. The applied similarity measure in not affected by impulsive noise as the outlying pixels are not considered when calculating the measure *R* using Eq. (15).

In the RLSF filter design we neglect the topological distance between pixels, as we observed that this has little impact on the final results and such a procedure proved beneficial also in the well known NLM algorithm, which assumes a self-similarity of the image features, not to mention the fact that omitting the spatial components allows to speed up the algorithm. We also used the squared Euclidean distances instead of distances as in the original ROAD definition when designing the *R* measure, as in this way it is more sensitive to the outliers and easier to compute as taking the square roots can be discarded.

The RLSF technique can be incorporated into the Mean Shift algorithm. It is worth noticing that the mean shift vector can be computed using the RSM, and spatial coordinates of the output are contained in the vector $\varvec{\xi }_i^{'}$ calculated using the scheme defined in Eq. (16). Nonetheless, instead of directly comparing the pixels $\varvec{x}_j$ from the block $\mathcal {B}_i$ with its center pixel $\varvec{x}_i$, we compare them with the central small window $\mathcal {W}_i$ and calculate the weight as given by (17). In each iteration, like in the MS, the new values of the RGB channels $\varvec{\eta }_i^{'}$ and new position $\varvec{\xi }_i^{'}$ of the processed pixel $\varvec{x}_i$ are calculated and the processing block $\mathcal {B}_i$ is moved so that it matches the new output pixel position. Of course, for the shifting of the block the new coordinates have to be rounded to the nearest integer, using the $\lfloor \cdot \rceil$ operation. Then, the new central window $\mathcal {W}$ in the shifted block is determined and its center is replaced by the RGB values of the RLSF filter output defined in (16).

Accordingly, the main difference of the new filtering technique when compared to the Mean Shift is the adoption of a robust similarity measure and the replacement, in each iteration, of the center of the processing block by the RLSF filter output. Then, the iteration process is continued until convergence is achieved. In the final step, the RGB values of the original pixel $\varvec{x}_i$ are replaced by $\varvec{\eta }_i^{'}$ obtained using the RLSF, analogously to the classic MS technique. The structure of the new algorithm is shown using pseudocode in Alg. 2 and also explained in Fig. 2.

The replacement of the central pixel of the processing block (center of window $\mathcal {W}$) is advantageous as it smooths out the Gaussian noise component through weighted averaging and it is able to remove the outliers when they occupy the window center. Adopting this approach, the impulsive pixels do not influence the trajectory of the block center (as the outlying values are not considered by the robust similarity measure) until the stopping criterion is satisfied. This allows us to apply the MS procedure directly on the images contaminated with mixed Gaussian and impulsive noise and if needed to perform the final segmentation procedure as suggested in^103^.
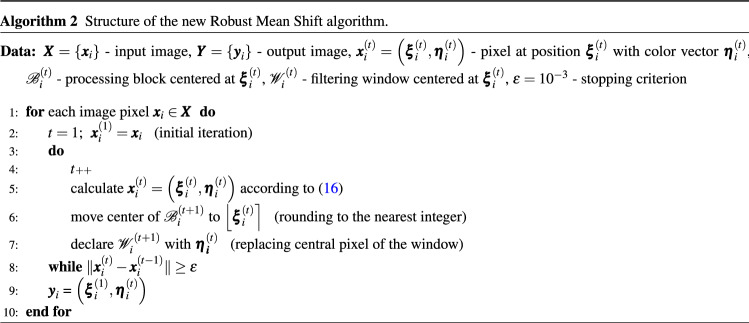

**Figure 1:**
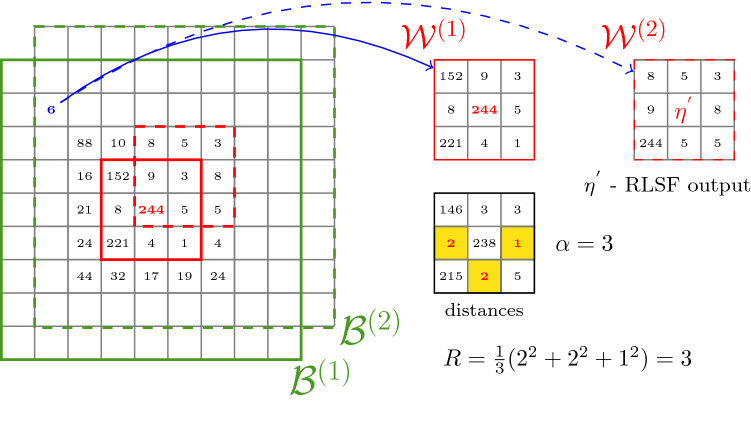
Illustration of two first steps of the Robust Mean Shift algorithm.

**Figure 2:**
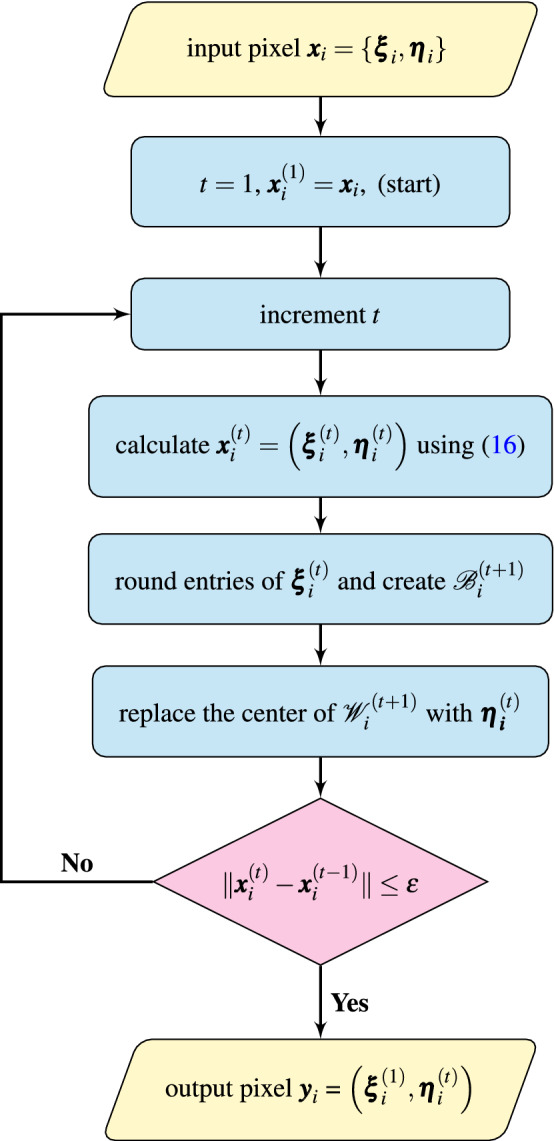
Diagram of the proposed algorithm showing how a given pixel $\varvec{x}_i$ is being processed: $\varvec{x}_i^{(t)} =(\varvec{\xi }_i^{(t)},\varvec{\eta }_i^{(t)})$ stands for the result of *t* iterations, (pixel at position $\varvec{\xi }_i^{(t)}$ with RGB vector $\varvec{\eta }_i^{(t)}$), the processing block and filtering window centered at $\varvec{\xi }_i^{(t)}$ are denoted as $\mathcal {B}_i^{(t)}$, $\mathcal {W}_i^{(t)}$ and $\epsilon =10^{-3}$ is used to establish the stopping criterion.

In order to better explain the proposed approach, Fig. 1 illustrates two first iterations of the Robust Mean-Shift algorithm. For simplicity, a grayscale image is used. The block and window indexes represent the number of iteration steps. In the first, a sample pixel of intensity 6 (marked blue) is compared to the central window. The three closest pixels intensities are 8, 5 and 4. For illustration, we assume that the new calculated processing block will be $\mathcal {B}_2$. This leads to a new central window $\mathcal {W}_2$ with the central pixel $\varvec{\eta }^{'}$ from the previous step (calculated using the RLSF) and unchanged pixels from the new processing block.

## Efficiency evaluation and comparison with existing filtering designs

**Figure 3:**
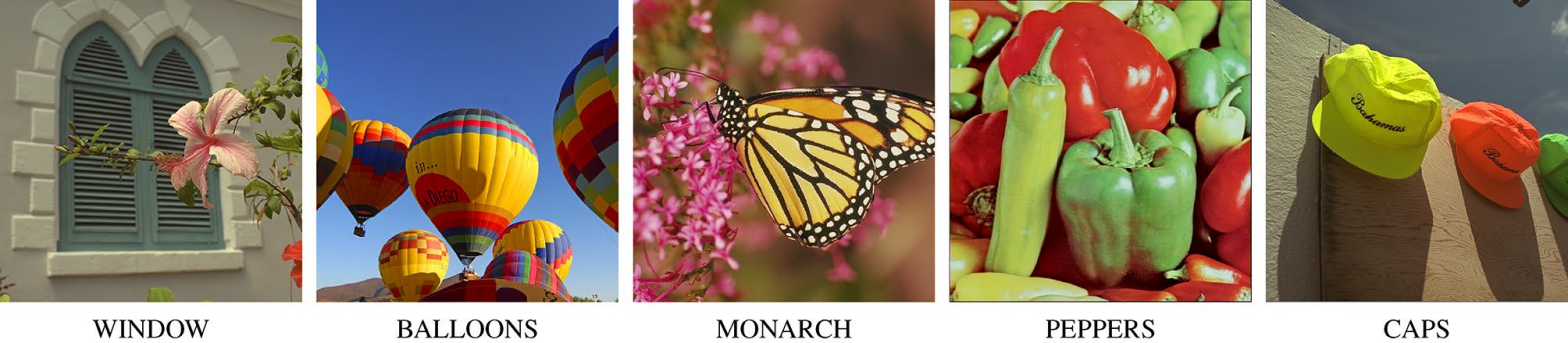
Color test images used in the evaluation of the new filter performance.

In an attempt to evaluate the efficiency of the proposed filter, two sets of color test images contaminated with different types of noise were prepared. Chosen test images are depicted in Fig. 3. The first set containing the mixed noise was firstly distorted by Gaussian noise with standard deviation in the range 10–50, (with step 10) and 10–50% of the pixels were subsequently replaced by random valued impulsive noise (with the same step), so that every RGB channel of a corrupted pixel was assigned a value drawn from a uniform distribution in the range [0, 255]. The second set of images was degraded only by random valued impulsive noise. To simplify the notation, noise level *p* denotes a Gaussian noise contamination with standard deviation *p* and with $p\%$ impulsive pixels. Such a combination of Gaussian and impulsive noise produces images with realistic levels of distortions and was used in our previous papers^7,8,104^, which enables us to compare the obtained results with those achieved using our other methods.

**Figure 4:**
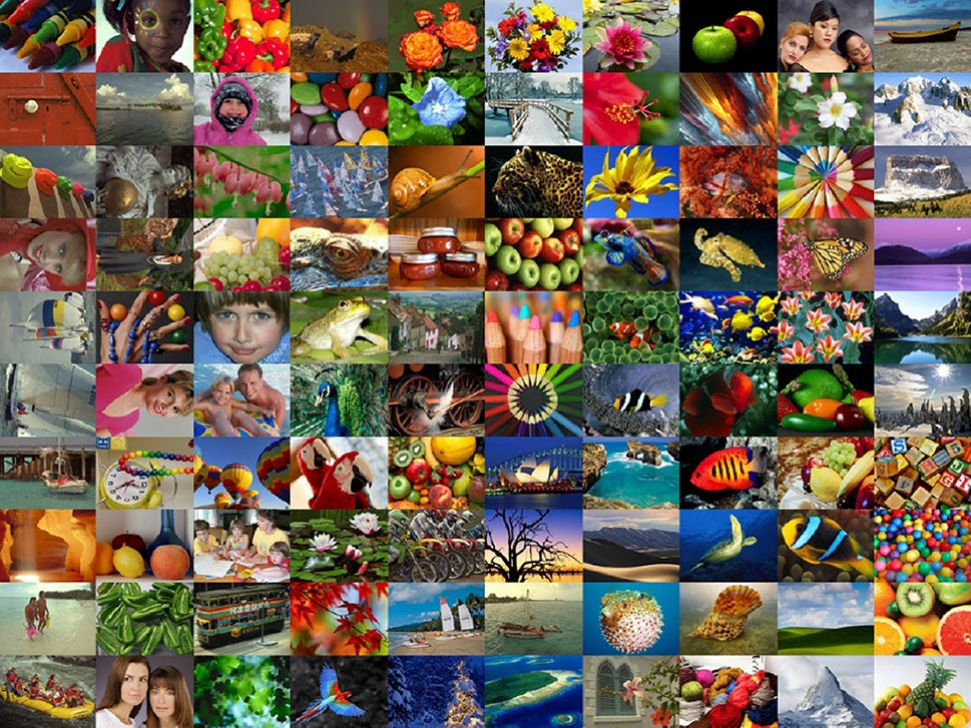
Dataset of color images used for the experiments.

The restoration efficiency has been assessed mainly using the commonly used PSNR and MAE quality measures^105–107^ defined as

18 $$\begin{aligned} \begin{aligned} \text {PSNR}=10\log _{10}\!\left( \frac{255^2}{\text {MSE}}\!\right) , \quad \text {MSE}=\frac{1}{3n} \sum _{i=1}^{n}\Vert \varvec{x}_{i}-\varvec{y}_{i}\Vert ^2, \end{aligned} \end{aligned}$$

where $\varvec{x}_{i}$ are the original image pixels, $\varvec{y}_{i}$ are the restored samples and the number of image pixels is *n*. In order to better express the ability of filtering out impulses, a *relaxed* Mean Squared Error measure ($\text {MSE}_{\text {R}}$) is used. The so-called Impulse Removal Index (IRI), is defined as^108^

19 $$\begin{aligned} \begin{aligned} \text {IRI} = 10\log _{10}\left( \frac{255^2}{\text {MSE}_{\text {R}}}\right) , \quad \text {MSE}_{\text {R}}=\frac{1}{3n}\sum _{i=1}^{n}\,\min _{\varvec{x}_j\in W_i} \Vert \varvec{y}_i -\varvec{x}_j\Vert ^2, \end{aligned} \end{aligned}$$

where $\varvec{x}_j$ are the original (clean) pixels contained in the filtering window $\mathcal {W}_i$ at image position *i* and $\varvec{y}_i$ is the filtering output.

Thus, IRI measures the deviation between the processed pixel and the set of original pixels located in the corresponding operational window of size $3\!\times \!3$. If the pixel is corrupted by impulsive noise, which is not removed, then the IRI will grow markedly. However, IRI will not increase when the corrupted pixel is replaced by a pixel close to one of its noise-free neighbors. In consequence, the IRI is a reliable measure of the ability of a filter to detect and replace corrupted picture elements. Additionally, the IRI measure does not penalize shifts of pixels making the edges sharper, which does not occur when using PSNR.

Furthermore, the Structural similarity (SSIM) measure and the Multi-Scale Similarity (MSSIM) were used to better express the image restoration quality in consistency with subjective ratings^109,110^. We converted these quality metrics into a logarithmic form in order to better compare the enhancement results

20 $$\begin{aligned} \begin{aligned} \text {SSIM}_{\text {log}} = -10\log _{10}\left( 1-\text {SSIM}\right) , \quad \text {MSSIM}_{\text {log}} =-10\log _{10}\left( 1-\text {MSSIM}\right) . \end{aligned} \end{aligned}$$

Because, the SSIM measure works on gray scale images, the luminance of the images was calculated using the following conversion rule: $L = 0.299\text {R} + 0.587\text {G} + 0.114\text {B}$.

**Figure 5:**
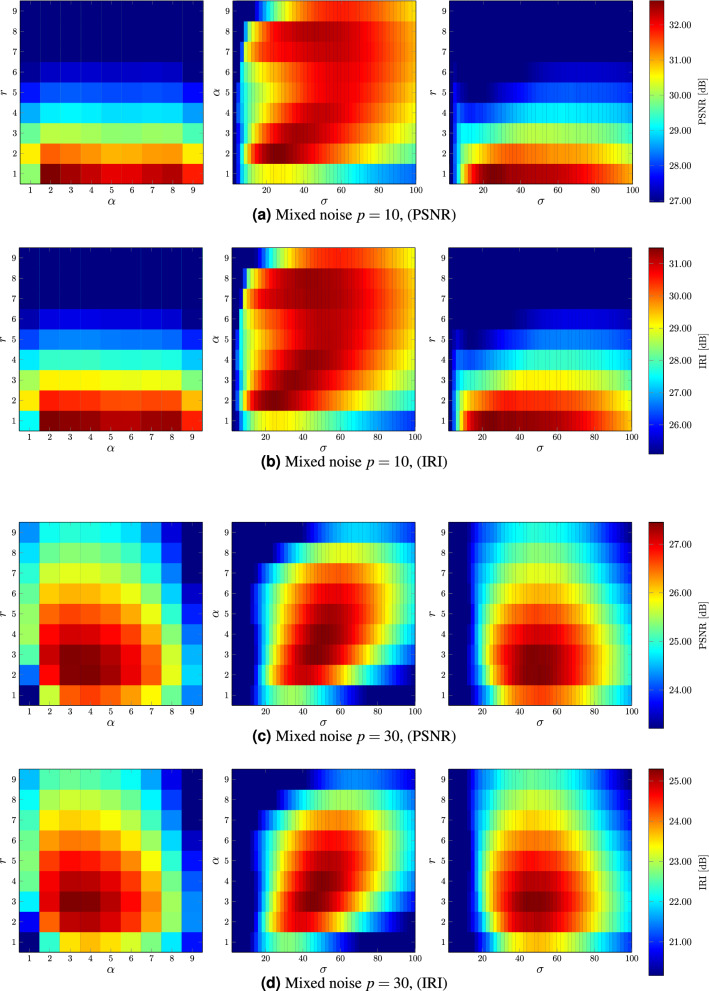
Dependence of highest achievable PSNR and IRI values on *r*, $\alpha$ and $\sigma$ parameters for image PEPPERS corrupted with mixed noise of intensity $p=10$ (**a**,**b**) and $p=30$ (**c**,**d**).

The filter efficiency has been tested on 100 color test images of resolution 640$\times$480, depicted in Fig. 4, available at http://denoising.net/ and also included in^6^, to determine the best tuning parameters of the new method. Initially, the influence of the processing block $\mathcal {B}$ size has been investigated. The plots in Fig. 5 show the dependence of the PSNR and the IRI measure on the radius *r* of the block $\mathcal {B}$ containing $(2r+1)^2$ pixels to tuning parameter $\sigma$ and also to the number of close neighbors $\alpha$. We chose an exemplary, well-known test color image PEPPERS contaminated at levels $p=10$ and $p=30$.

For low noise level, a processing block of the size of $$3\!\times \!3$$, ($$r=1$$), which coincides with the central window $$\mathcal {W}$$, is large enough, although for higher noise levels, a block of $$5\!\times \!5$$, ($$r=2),$$ gives much better results. The choice of the second parameter—$$\alpha $$ does not influence significantly the quality measures and the three nearest pixels ($$\alpha =3$$) are optimal both for low and high contamination levels. The third parameter $$\sigma $$ is not dependent on both *r* and $$\alpha $$, but depends on the noise level. It reduces the impact of Gaussian noise in the filtering process, thus the higher the Gaussian noise component is, the higher the $$\sigma $$ parameter is needed. The recommended value of $$\sigma $$ for low noise level is 30 and for higher intensity $$\sigma =50$$.

In Tables 1, 2, 3, 4 the new filtering design has been compared with a set of filters used for the reduction of noise in color images using the PSNR, IRI, SSIM and MSSIM restoration quality measures. The following filters were chosen for comparisons:

- Mean-Shift Filter, (MS)^85^,
- Quick-Shift Filter, (QS)^99^,
- Medoid-Shift Filter, (MEDS)^100^
- Bilateral Filter, (BF)^87^,
- Non-Local Means Filter, (NLM)^16^,
- Vector Median Filter, (VMF)^111^,
- Block-Matching and 3D filtering, (BM3D)^17^,
- Patch-based Approach to Remove Impulse-Gaussian Noise, (PARIGI)^112^,
- Weighted Encoding with Sparse Nonlocal Regularization, (WESNR)^71^,
- TV-based restoration method with $\ell _0$TV-norm data fidelity, ($\ell _0$TV)^76^,
- Guided Bilateral Filter, (GBF)^113^.

For the MS filter, we tested three variants which treat in a different way the weight assigned to the central pixel of the processing block. As for the central pixel of the block the similarity to itself is considered, the assigned weight always takes on the maximum value 1, independently of the pixel RGB channels and the block structure. When this pixel is corrupted, then, even though its role in the averaging process should be diminished, it is still considered as deserving the highest weight, which makes that the impulses are mostly retained. The only way to force the algorithm to decrease the influence of the central pixel of the block is to set a high value of the parameter $\sigma _{\text {r}}$, which, however causes a considerable blurring. Therefore, to make a valid comparison of the new ROMS filter with the MS, its three versions have been evaluated^114,115^

- $\text {MS}_{\text {STD}}$—standard version, as described in^85^, considering all pixels in the filtering block, (including the central pixel),
- $\text {MS}_{\text {NC}}$—the central pixel of the block is omitted in the calculations of the weights in Eq. (17), (no central pixel considered),
- $\text {MS}_{\text {MAX}}$—the maximum weight calculated for the pixels in the block (excluding the central pixel) is assigned^114^.

The analysis of the PSNR results summarized in Table 1 indicates that the ROMS filter has much better denoising capabilities than the Mean Shift and its 3 modifications regarding the treatment of the central pixel. The performance of the MS modifications does not differ significantly in terms of PSNR and IRI, both for low and high contamination. However, when analysing the results in terms of the SSIM and MSSIM measures, which better express the perceived quality of the restored images, the version $\text {MS}_{\text {NC}}$ is superior to the standard MS implementation. In addition, the Quick Shift and Medoid Shift were not able to effectively suppress the mixed noise, although the latter proved to be generally more effective when analyzing the PSNR results. Similar conclusions can be drawn while analyzing the results in terms of the IRI (Table 2), SSIM and MSSIM measures (Tables 3, 4).

Analyzing the obtained results presented in the Tables, the proposed denoising scheme outperforms other filters taken for comparison when the test images were distorted by mixed noise of high intensity. For lower contamination levels, only the WESNR filter achieved for some images slightly better performance. The IRI results show that the ROMS filter efficiently removes the impulsive noise outperforming other methods. The satisfying properties of the ROMS are also confirmed when scrutinizing the results in terms of SSIM and MSSIM.

The very satisfying performance of the ROMS filters expressed using the objective quality measures can be also confirmed visually. Figures 7 and 8 show that the ROMS filter can cope, both with the pure impulsive and mixed noise, and it exhibits significantly better denoising efficiency than the competitive methods. The impulsive noise is much better attenuated, and the Gaussian component is efficiently reduced. The image details are well preserved, and the edges are even sharper than in the undistorted test images.

The restoration results obtained using the WESNR show that the filter is prone to generate color artifacts, especially for high contamination levels. The enhancement results achieved using the PARIGI filter reveal that this filter tend to retain impulsive pixels and is not able to suppress the Gaussian noise for high contamination levels.

It can be also observed that the standard Mean Shift is forced to increase the smoothing parameter to suppress the impulses, which makes the images blurry. The QS and MEDS techniques produce much sharper images, however the edges are jagged and exhibit a strong zipper effect. In contrast, the proposed robust modification does not experience any problems with the outliers injected by the noise process and is able to sharpen image edges, which is well expressed by the IRI measure. The visual comparison of the images presented in Figs. 7a) and 8a) shows that the proposed Robust Mean-Shift offers very satisfying image enhancement results when only impulsive noise is present, what makes this algorithm very versatile. The effectiveness of the proposed technique is also confirmed in Fig. 11, which presents the enhancement results of real noisy images depicting 2 works of art and also a cDNA microarray^116^. As can be observed the noise of unknown characteristic is well suppressed and edges are sharpened.

The ability of the proposed algorithm to sharpen edges of the image objects is very beneficial when they are to be segmented or edge detection is to be performed. Therefore, we decided to evaluate the efficiency of ROMS to enhance image edges. To that end, we prepared a test color image SQUARES consisting of square areas with slowly varying colors depicted in Fig. 9. The position of the edges was known, so we could objectively evaluate the ability of the ROMS and other filters to restore them.

The SQUARES image was corrupted with impulsive and mixed noise of different intensity and restored using ROMS, MS, QS, MEDS and other filters used for comparisons. For the objective assessment of the edge preservation ability of the tested algorithm, the well-known Pratt’s Figure of Merit (FOM)^117^ was used.

This measure is defined as follows

21 $$\begin{aligned} \text {FOM} = \frac{1}{\max (I',I)}\sum ^{I'}_{i=1} \frac{1}{1+\gamma \, d_i}, \end{aligned}$$

where *I* and $I'$ are the numbers of detected edge pixels in the clean and filtered images, $d_i$ is the topological distance between the found edge pixel and its closest pixel belonging to the set of ideal edges. Additionally, a design constant $\gamma =9$ was used to penalize displaced edges. The FOM values are in the interval [0, 1] and higher values indicate better edge detector performance.

Figures 9a) and 10a) show the test image SQUARES distorted with impulsive and mixed noise together with the result of their enhancement. As can be noticed, the restoration quality excels the Mean Shift and its modifications (MEDS and QS) both in the case of impulsive and mixed noise. The ROMS filter removes efficiently impulsive noise, the edges are not distorted by the zipper effect and no blotches are produced.

The obtained results also exhibit the tendency of the WESNR to produce color blotches both for impulsive and mixed noise. The inability of the PARIGI filter to suppress the strong Gaussian noise was also confirmed.

The very good edge-enhancing properties of ROMS are supported by the maps of the Vector Range edge detector^118,119^ presented in Figs. 9b) and 10b). The edges are continuous and do not deviate much from those obtained using the undistorted image which is reflected by the FOM measures summarized in Table 5. The analysis of the FOM values show that the ROMS considerable excels all competitive techniques when the ability to suppress impulsive and mixed noise in flat areas and to retain their edges is considered.

**Figure 6:**
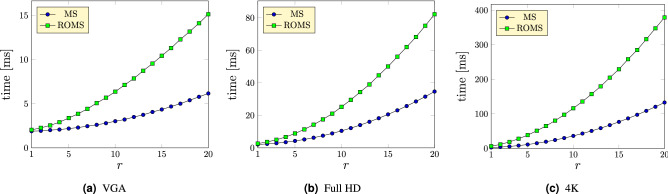
Comparison of computational efficiency of ROMS with the MS filter for varying block radius *r* and $\alpha =3$ on test images with VGA, Full HD and 4 K resolutions.

Figure 6 shows the comparison of the execution time of the ROMS filter when compared with the standard MS. The proposed filter is slower than MS, but still enables its application in real-time processing tasks or for video denoising even for images in full HD resolution. The experiments have been performed on a CUDA compatible NVIDA RTX2080Ti graphics card. The plots showing the dependence of the execution times on block radius *r* were obtained averaging the results of 1000 repetitions.

## Conclusions

In the paper a Robust Mean Shift technique, able to efficiently suppress mixed Gaussian and impulsive noise, has been presented. The new filtering framework makes use of a robust similarity measure which compares a pixel belonging to a processing block with a given number of closest samples in a filtering window in its center. Thereby, the introduced similarity measure is resilient to noise disturbances and allows to construct robust noise filtering designs.

The adoption of the Mean Shift concept proved to deliver very satisfying noise restoration results, even for images degraded by impulsive and mixed noise of high intensity. The obtained results were presented using objective quality measures and indicated that the new filter outperforms the Mean Shift and also Medoid Shift and Quick Shift methods, which are its modifications developed to make the algorithm faster.

The proposed ROMS excels some widely used denoising methods, like BF, NLM, BM3D or VMF, which are utilizing the Euclidean distance, in terms of standard restoration quality metrics. The proposed robust similarity measure between a pixel and a patch in the center of the processing block allows to substantially diminish the influence of the impulsive pixels. In this way, the susceptibility of the Euclidean distance to outliers can be considerable decreased, which enables to construct new methods based on the classical, well-researched algorithms.

The proposed method is especially effective for images contaminated with mixed noise of high intensity. As the pixels from the filtering block are compared with the patch of pixels in its center, some tiny details are treated as noise and removed. This can be seen as a drawback when the preservation of tiny details in images with low contamination is desired. Another feature of the proposed filter is its tendency to sharpen image edges. Generally, edge enhancement is beneficial in most applications, however the creation of too strong edges can lead to image oversegmentation. To decrease the impact of this effect, the filter parameter $\sigma$ can be increased, which leads to softer edge profiles without diminishing the efficiency of the filter to suppress the mixed noise.

The visual analysis of the experiments performed on a set of standard color images proved that the new filter can efficiently remove the impulsive noise while smoothing out the Gaussian noise component. The beneficial feature of the new filtering design is its ability to preserve and even enhance the image edges. An analysis performed applying the Vector Range edge detector and the Pratt’s Figure of Merit (FOM) revealed that the new technique is able to precisely locate the edges and significantly outperforms the designs based on the Mean Shift and its modifications and also other competitive denoising techniques.

The described method can be used for the enhancement of both gray scale and color images and their temporal sequences. It can be easily extended to work on multispectral images corrupted by a mixture of Gaussian and heavy tailed noise. Possible applications include underwater imagery, virtual enhancement of works of art, restoration of old photographs, filtering of microscopy images and enhancement of multispectral satellite, astronomical and medical data acquired from various modalities. Additionally, the elaborated approach can be applied for image segmentation tasks and also for multi-dimensional data clustering^120^.

Future work will be focused on the incorporation of the robust pixel to patch similarity measure to the structure of the Medoid and Quick Shift, so that they will be able to cope better with impulsive and mixed noise corrupting color images. Preliminary experimental results show that the adoption of an additional weight assigned to the pixels from the processing block, expressing their degree of corruption, considerable improves the denoising effectiveness of the developed filtering framework. In this way, a new family of very fast filters utilizing the Medoid and Quick Shift concept will be elaborated.

**Table 1:**
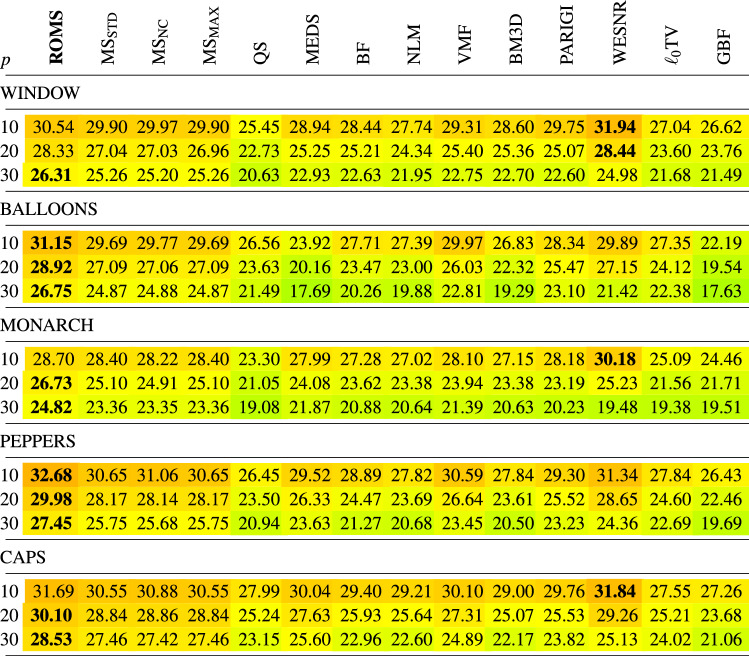
Comparison of the PSNR quality measure of the restored color test images contaminated with mixed noise using the new ROMS filter and state-of-the art denoising methods.

**Table 2:**
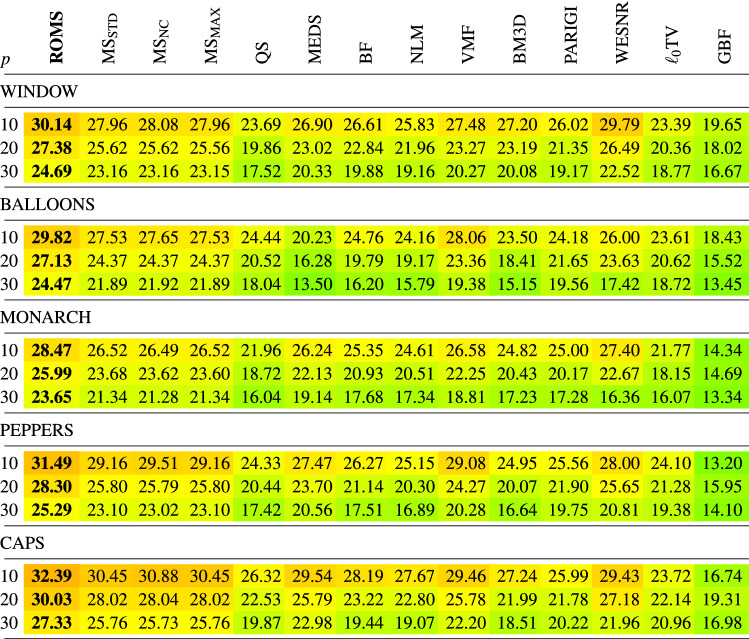
Comparison of the IRI quality measure of the restored color test images contaminated with mixed noise using the proposed ROMS filter and state-of-the art filtering methods.

**Table 3:**
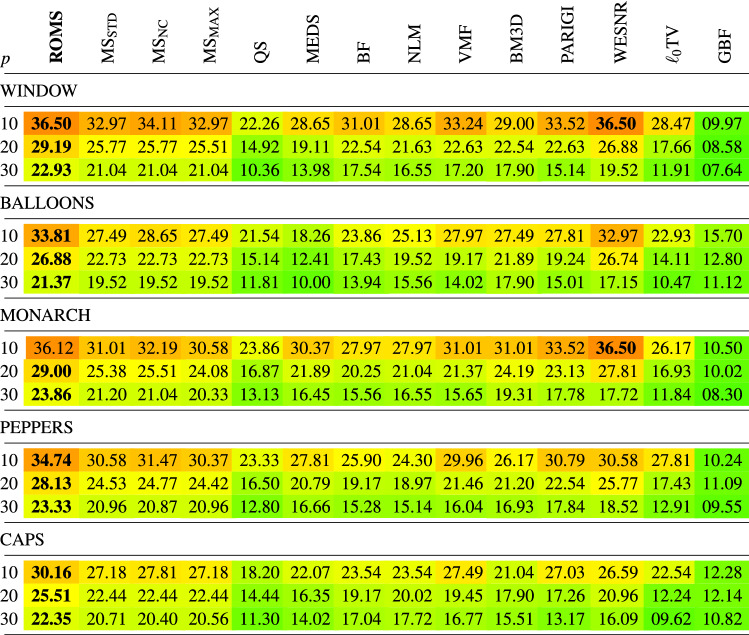
Comparison of the SSIM quality measure of the restored color test images contaminated with mixed noise using the proposed technique and state-of-the art filtering methods.

**Table 4:**
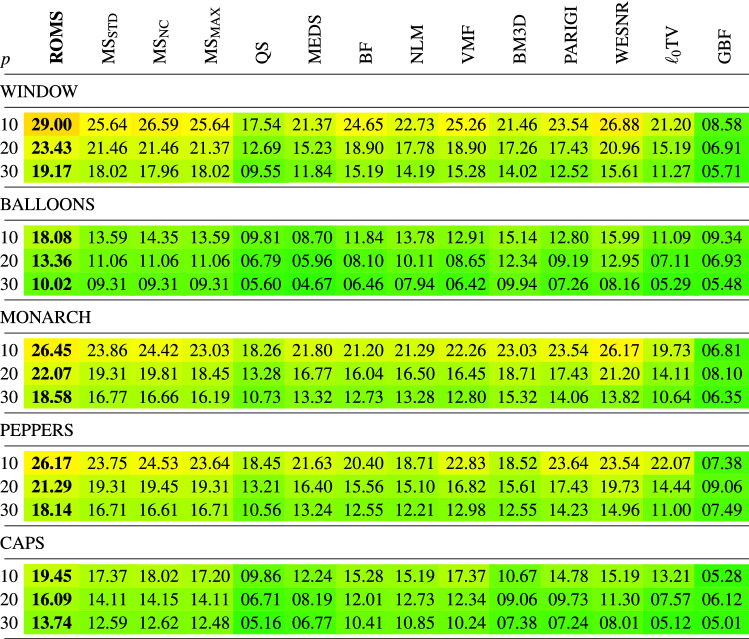
Comparison of the MSSIM quality measure of the restored color test images contaminated with mixed noise using the proposed ROMS technique and state-of-the art filtering methods.

**Table 5:**
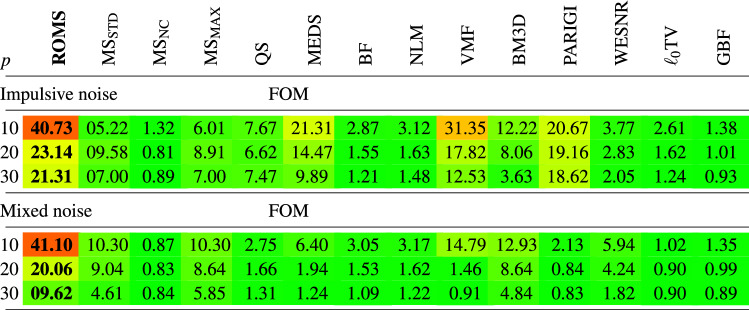
Comparison of the FOM edge restoration quality measure of the SQUARES test image contaminated with impulsive and mixed noise when filtered with the new ROMS technique and competitive filters.

**Figure 7:**
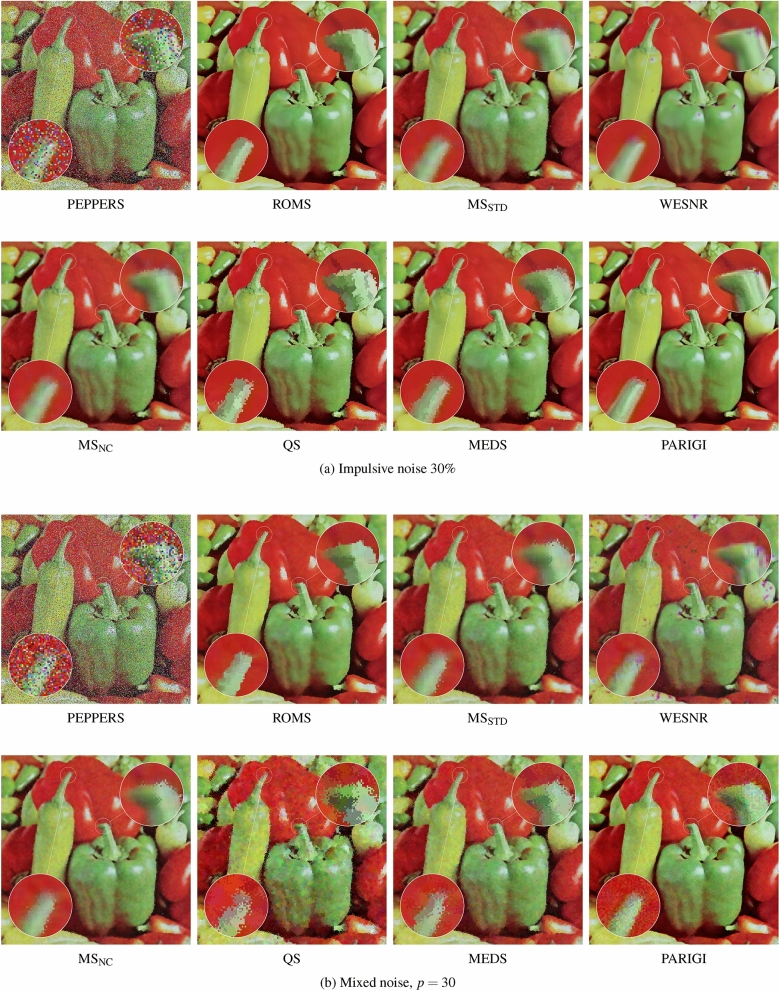
Efficiency comparison of the the ROMS algorithm with the competitive techniques evaluated on test image PEPPERS contaminated with impulsive noise of $30\%$ (**a**) and with mixed noise of intensity $p=30$ (**b**).

**Figure 8:**
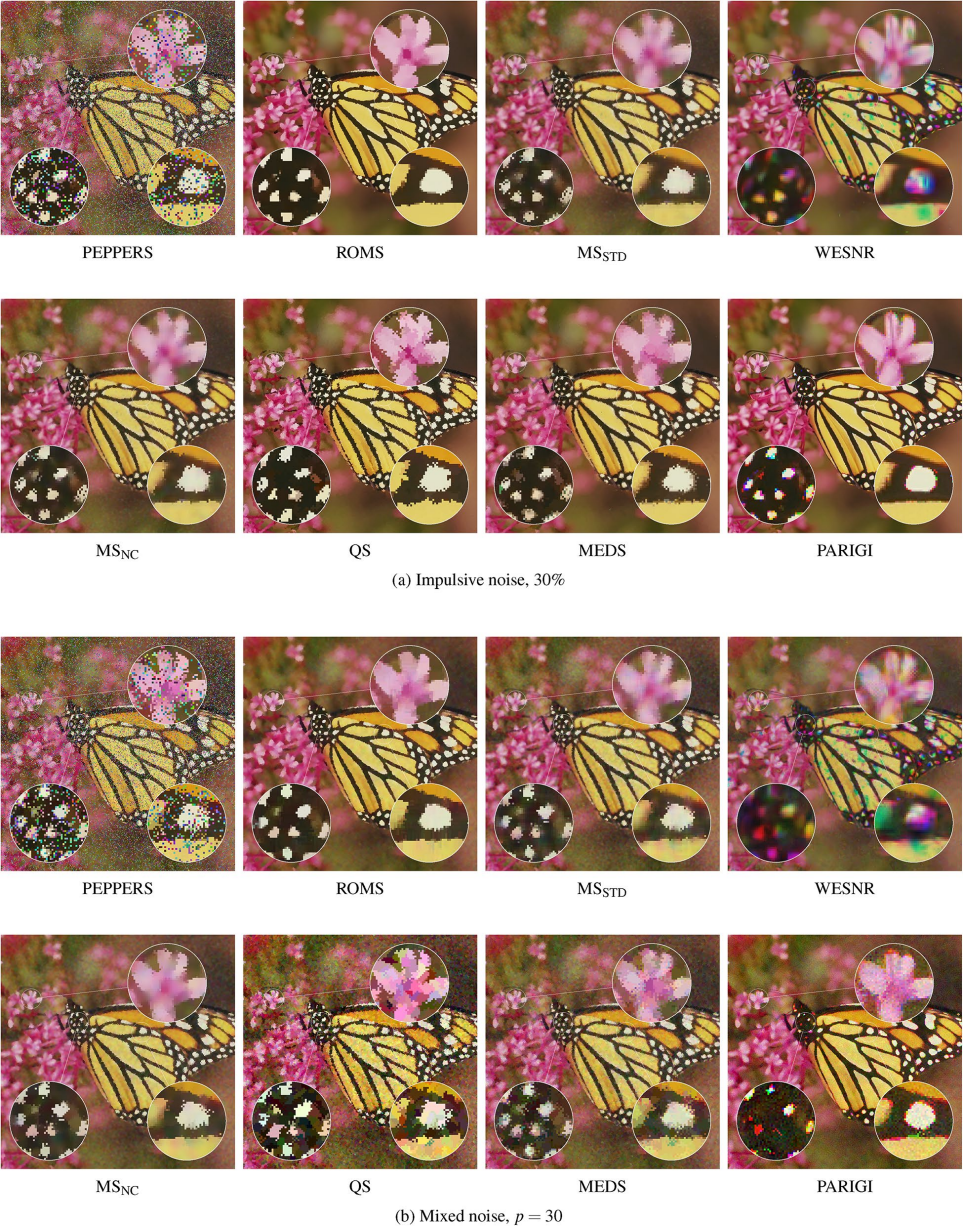
Efficiency comparison of the the proposed ROMS algorithm with the MS technique on MONARCH images contaminated with impulsive noise of $30\%$ (**a**) and with mixed noise of intensity $p=30$ (**b**).

**Figure 9:**
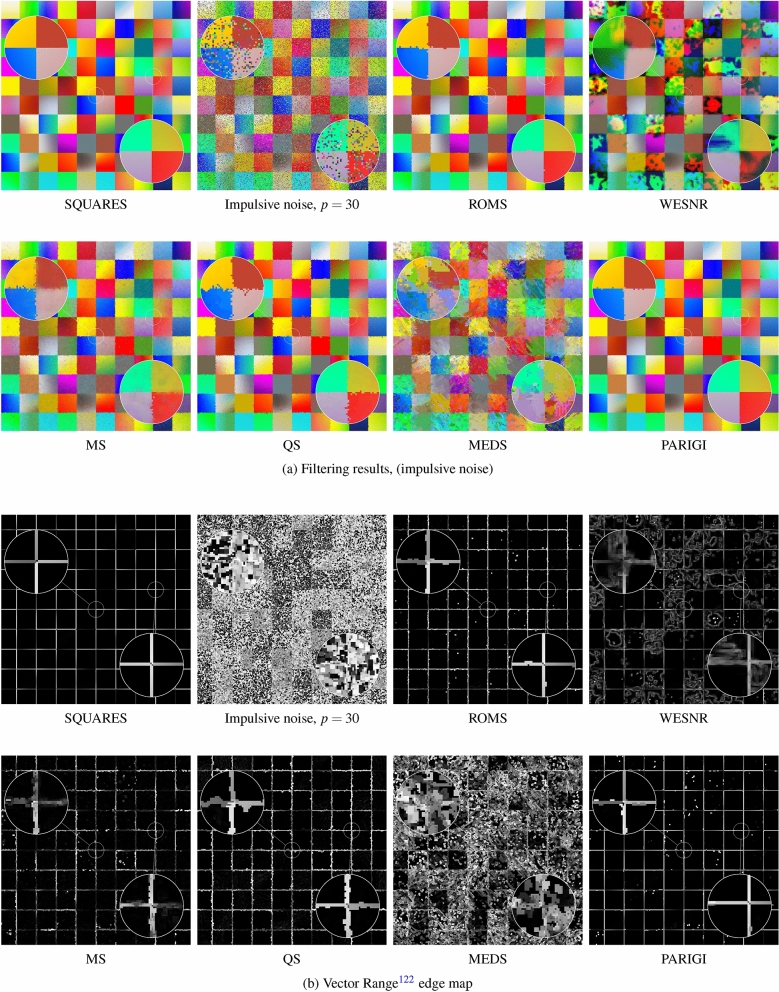
Efficiency comparison of the the proposed ROMS with MS and its fast modifications using the SQUARES test image contaminated with impulsive noise $p=30\%$.

**Figure 10:**
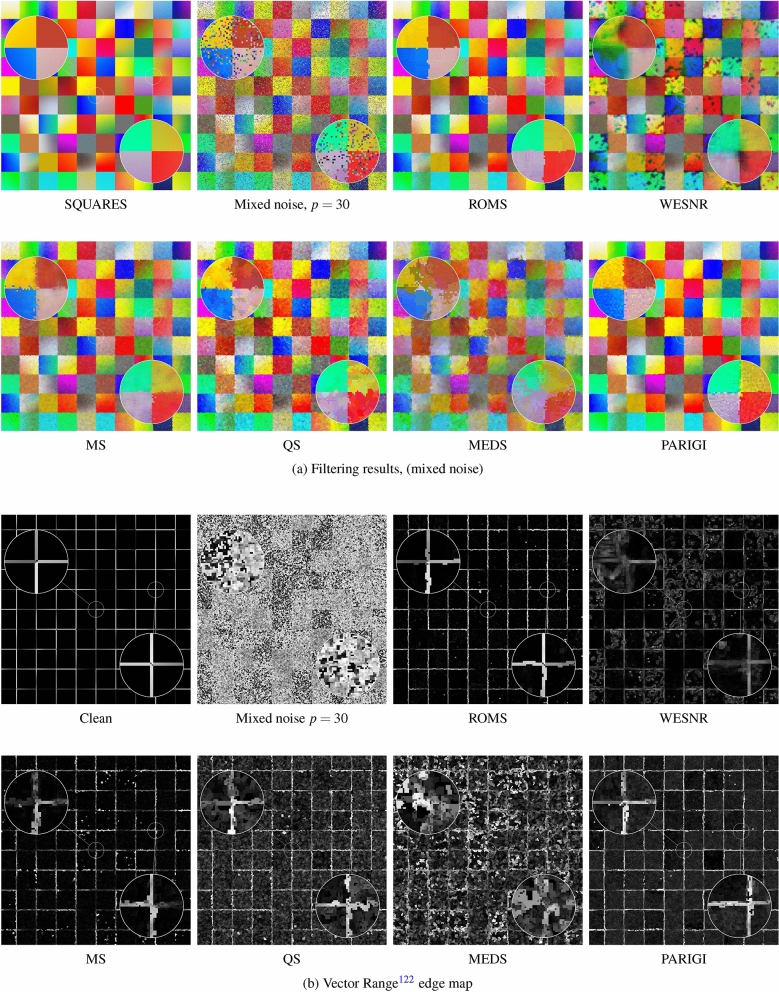
Efficiency comparison of the the proposed ROMS with MS and its fast modifications using the SQUARES test image contaminated with mixed noise $p=30$.

**Figure 11:**
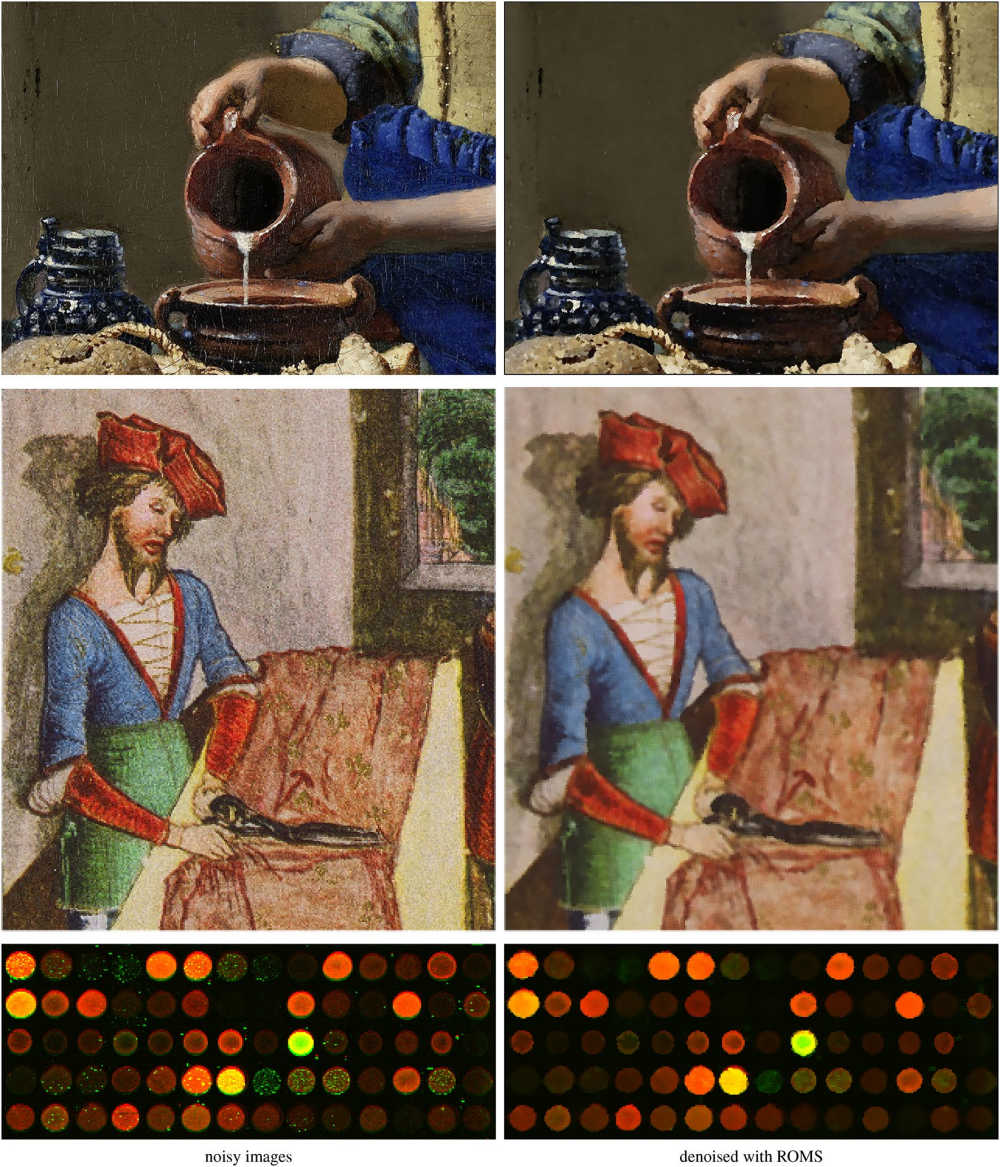
Filtering results of the proposed ROMS using real noisy images. From top to bottom: cropped and zoomed part of the painting “The Milkmaid” by Johannes Vermeer, a miniature from the Balthasar Behem Codex and a cDNA microarray^116^. The images were processed using the parameters: $\alpha =3$, $\sigma =30$ and $r=1$.

## Data Availability

Algorithm implementation with working code of this work is available under https://github.com/dkusnik/RMS.
